# Design of MnO_x_/TiO_2_ Nanostructures for Photocatalytic Removal of 2,4‐D Herbicide

**DOI:** 10.1002/open.202400154

**Published:** 2024-12-13

**Authors:** Angeles Mantilla, Sandra Cipagauta Díaz, Enrique Samaniego Benitez, Francisco Javier Tzompantzi Morales, Michelle Navarrete Magaña

**Affiliations:** ^1^ Advanced Technology Instituto Politécnico Nacional CICATA-Legaria Legaria 694 Col. Irrigación 11500 Ciudad de México; ^2^ Chemistry Deparment Universidad Autónoma Metropolitana-Iztapalapa Av. San Rafael Atlixco 186 Leyes de Reforma 09340 Ciudad de México; ^3^ Avanced Technology CONAHCyT-Instituto Politécnico Nacional CICATA-Legaria Legaria 694 Col. Irrigación 11500 Ciudad de México; ^4^ Chemistry Academies Instituto Politécnico Nacional UPIICSA Av. Té 950 Granjas México 08400 Ciudad de México

**Keywords:** Sol-gel processes, Photocatalysis, Nanostructures, Photooxidation, Reaction mechanisms

## Abstract

The research and modification of semiconductors through different synthesis routes allow obtaining materials with optimal properties to induce new energy levels and improve charge separation efficiency. In this context, the sol‐gel method was used to synthesize TiO_2_‐based materials doped with different percentages of MnO_x_ to evaluate their photocatalytic activity in the degradation of the herbicide 2,4‐dichlorophenoxyacetic acid (2,4‐D) in water under UV irradiation. Characterization results revealed a reduction in crystallite size to 7.2 nm. Adding MnO_x_ enhanced the optical absorption of TiO_2_, resulting in a shift toward the red end of the spectrum of the forbidden energy band. The photocatalytic activity increased significantly with the percentage of MnO_x_, reaching a maximum degradation of 70 % in 6 hours with the 3 MnTi material. This increase was attributed to the synthesis method, which facilitated the formation of nanostructured heterojunctions mainly composed of TiO_2_ and MnO_2_, reducing the recombination of electron‐hole pairs. TEM analysis confirmed these structures. A reaction mechanism for the 3 MnTi material is proposed, considering the mobility of charge carriers and the photooxidation processes of the pollutant.

## Graphical abstract

This study addresses the synthesis and characterization of MnO_x_/TiO_2_ materials by the sol‐gel method to evaluate their efficacy in the degradation of 2,4‐D in aqueous solution. The findings indicate that the incorporation of 3 %wt MnO_x_ in TiO_2_ significantly enhances the degradation of the herbicide, a process mainly attributed to the participation of superoxide radicals and holes as oxidizing species.

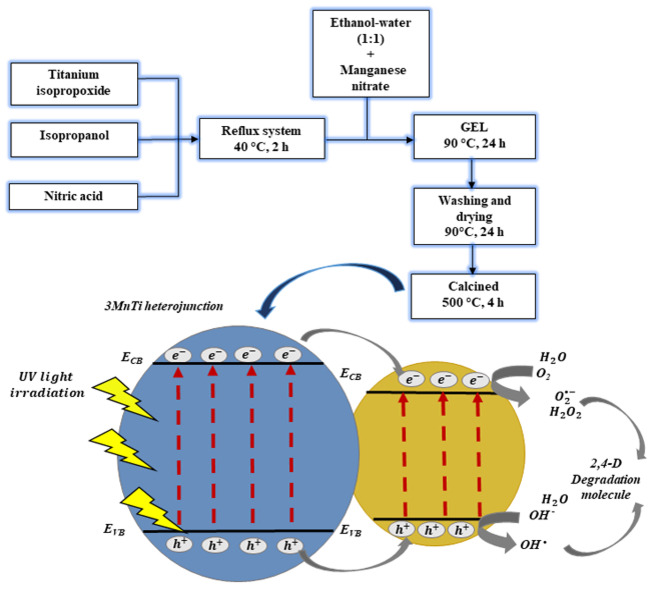

## Introduction

Nowadays, the availability of drinking water is one of the most significant challenges. Ensuring that water resources are contamination‐free has become increasingly important, given the substantial expansion of population and growing food demands.^[1]^ The indiscriminate use of herbicides in agriculture has led to excessive contamination of aquifers and groundwater.[^2^, ^3^] Within this category of compounds, 2,4‐dichlorophenoxyacetic acid (2,4‐D) stands out as a widely used herbicide worldwide, commonly applied in pest management.^[4]^ 2,4‐D can induce severe gastrointestinal, respiratory, and dermatological disorders.[^5^, ^6^] It is, therefore, imperative to address the treatment of these contaminants in water. The use of photocatalysts for water purification has attracted attention due to its applicability to a wide variety of pollutants.[^7^, ^8^, ^9^, ^10^] Advanced Oxidation Processes (PAO's) are technologies that reduce the toxicity of resistant compounds and their partial or total elimination.[^11^, ^12^, ^13^, ^14^] These technologies involve a semiconductor that could be excited by light with an energy higher than its band gap to produce electron and hole pairs, which produce highly oxidizing species, such as ⋅OH radicals and superoxide radicals.[^15^, ^16^, ^17^] Titanium dioxide (TiO_2_) is mostly described as a versatile and effective photocatalyst in the oxidation of different emerging pollutants in the presence of oxygen due to its essential properties such as excellent oxidation potential and photostability; it is economical and non‐toxicity. However, the significant factors affecting TiO_2_ photocatalytic performance are pore volume, size, specific surface area, and band gap energy.[^18^, ^19^] Another major disadvantage of TiO_2_ is its high rate of recombination of photo‐generated electron‐hole pairs (e^−^/h^+^), which decreases the quantum yield and promotes a low transfer efficiency of one electron to oxygen that limits the photooxidation of organic contaminants.^[20]^ In order to overcome these issues, doping using metallic, non‐metallic, and oxide prossessing, a narrow band gap is implemented leading to benefits such as reducing the energy gap of TiO_2_ and improving photocatalytic efficiency. Within these strategies, the inclusion of metal oxide semiconductors stands out as the most promising. Previous research has evidenced the synergistic effect of coupling two semiconductors for the formation of heterostructures, which gives unique properties to the material. This phenomenon leads to an improvement in the proportion of active sites on the surface, an efficient separation of charge carriers, and a modification in the structure of the energy levels.^[21]^ In this sense, manganese oxide (MnO_x_) has stood out due to different factors, including rich structures and morphologies, natural abundance, various valence states, low price and non‐toxicity to the environment, and high thermal stability.[^22^, ^23^, ^24^, ^25^, ^26^] The base unit of MnO_2_ consists of an octahedral structure formed by one Mn atom and six O atoms. Additionally, it presents five different crystalline phases: α‐, β‐, γ‐, δ‐ and λ‐MnO. The crystalline structure of MnO_2_ can substantially impact the photocatalytic activity through different synthetic processes, in which the α‐MnO_2_ phase presents the highest activity.[^27^, ^28^, ^29^] Several researchers have reported the study of semiconductor materials based on MnO_2_/TiO_2_ for their application in decontamination processes. Elham et al.^[30]^ prepared N‐TiO_2_/MnO_2_ nanocomposite for gaseous formaldehyde degradation. Qi et al.^[31]^ prepared MnO_2_/TiO_2_ nanobelts for toluene degradation under vacuum UV irradiation. Based on the literature reviewed, the MnO_2_/TiO_2_ type materials present ideal photocatalytic properties for this research, such as the different oxidation states of Mn, which will allow the modification of the TiO_2_ crystalline structure and thus improve the processes of charge separation, as well as its efficiency in the photocatalytic degradation of the 2,4‐dichlorophenoxyacetic acid (2,4‐D) herbicide in an aqueous medium.

## Results and Discussion

**Figure 1:**
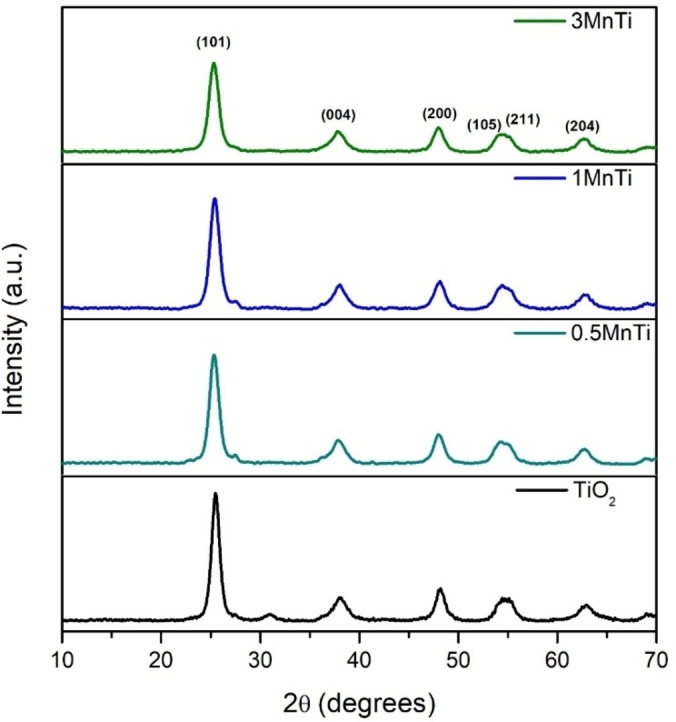
X‐ray diffraction for TiO_2_ and MnTi heterostructures synthesized by the sol‐gel method.

The identification of the crystalline phases, as well as the lattice parameters and crystallite size in the TiO_2_ and MnTi (0.5, 1 and 3 %wt) materials calcined at 500 °C were obtained from the diffractograms shown in Figure 1.

It was observed that the TiO_2_ sol‐gel presents diffraction peaks at 2θ=25.3°, 37.9°, 47.8°, 54.6° and 62.8°, corresponding to planes (101), (004), (200), (105) and (204) of the anatase phase with tetragonal crystal system (JCPDS No. 21–1272).^[32]^ To calculate the crystallite size, the diffraction peak at 2θ=25.3° associated with the reflection plane (101) was used using the Scherrer equation.^[33]^ The results obtained are listed in Table 1. The diffractograms of the MnTi materials presented characteristic peaks of the anatase phase of TiO_2_; however, reflection lines associated with MnO_2_ were not detected, probably due to a low mass of MnO_2_ in the sample and to the high dispersion of manganese oxide into titanium oxide.^[19]^ On the other hand, an increase in the average width of the peak associated with the plane (101) of the anatase phase was observed. The average peak width is inversely proportional to the value of the crystallite size, which was calculated by means of the Debye‐Scherrer Scherrer equation.^[33]^ The values obtained are summarized in Table 1. As can be seen, the crystallite size decreased as a function of the percentage of MnO_x_ increases, obtaining values between 9.0 and 7.2 nm. This result is due to during the synthesis process, when the manganese was added to the solution, it is very likely that a small amount of the MnO_x_ would remain on the surface of the TiO_2_, slowing down the growth of the crystalline network and, consequently, the size of the crystals.^[34]^

**Table 1:** Lattice parameters and crystallite size of TiO_2_ and MnTi nanostructures.

Sample	Lattice parameters (Å)		Crystallite size (nm)
	a=b	c	
iO_2_	3.78	9.30	9.0
0.5 MnTi	3.77	9.30	7.9
1 MnTi	3.77	9.40	7.7
3 MnTi	3.77	9.53	7.2

**Figure 2:**
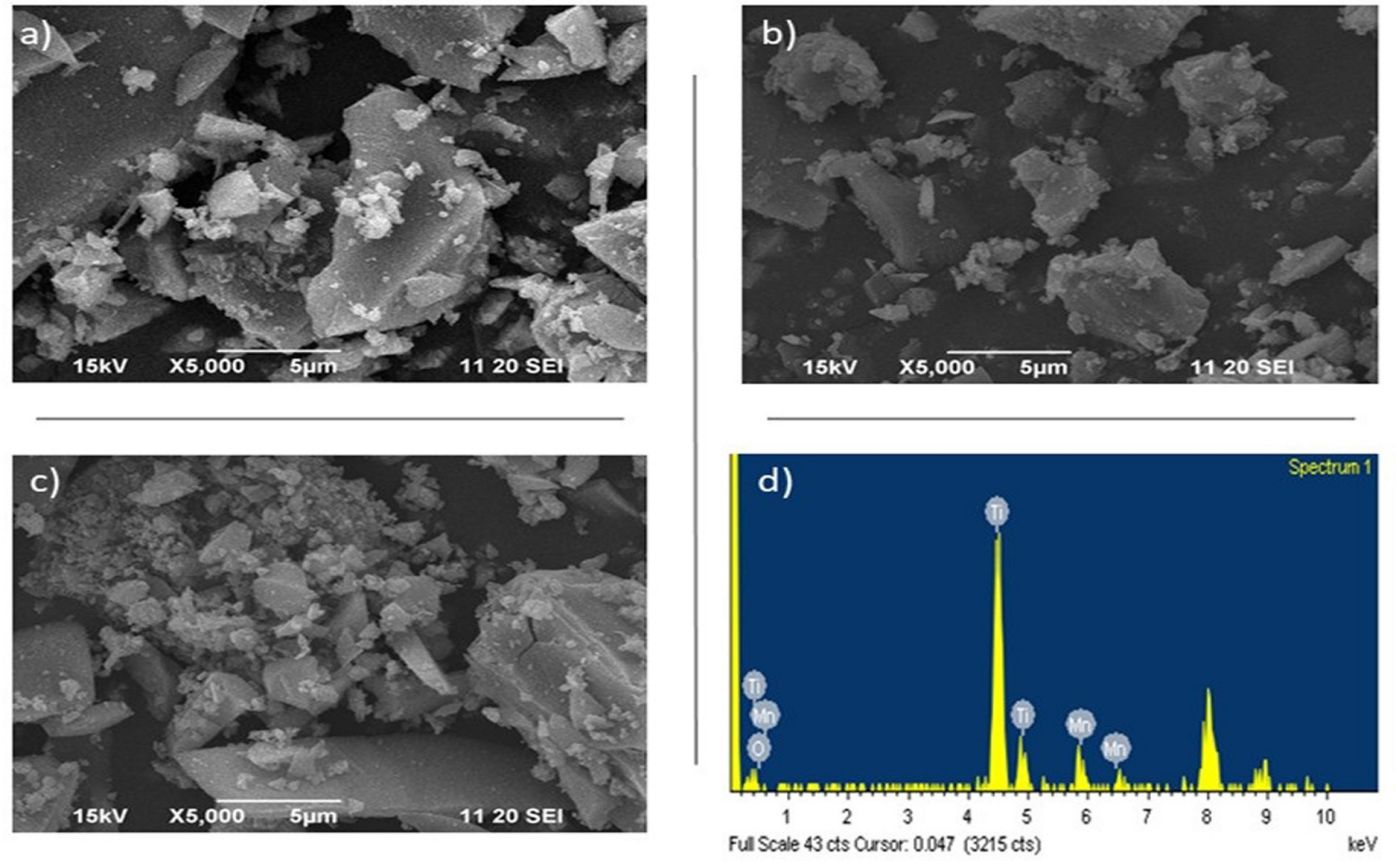
Scanning Electron Microscopy (SEM) image of a) 0.5 MnTi, b) 1 MnTi and c) 3 MnTi nanomaterials, and d) EDS for 3 MnTi.

A Scanning Electron Microscopy (SEM) study was carried out in order to know the surface morphology obtained in the materials synthesized with MnO_x_. Figure 2(a–c) shows the SEM images for the 0.5, 1 and 3 MnTi photocatalysts, respectively. In Figure 2(a‐b), it can be seen that the materials present morphology with nano aggregates of irregular shape and size due to an agglomeration effect developed during the rapid hydrolysis reaction. On the other hand, the micrograph (Figure 2c) reveals that the sample is mainly composed of particles with different shapes, mostly faceted with a larger particle size with respect to the 0.5 and 1 MnTi nano‐structured materials.^[35]^ With the X‐ray energy dispersive spectroscopy (EDS), it was possible to verify the presence of Mn and TiO_2_ in all the samples doped with MnO_2_. As a reference, the EDS of the 3 MnTi sample is presented in Figure 2(d), showing the Mn peaks at 0.792 keV, O_2_ at 0.525 keV, and Ti at 0.452 keV, respectively.

**Figure 3:**
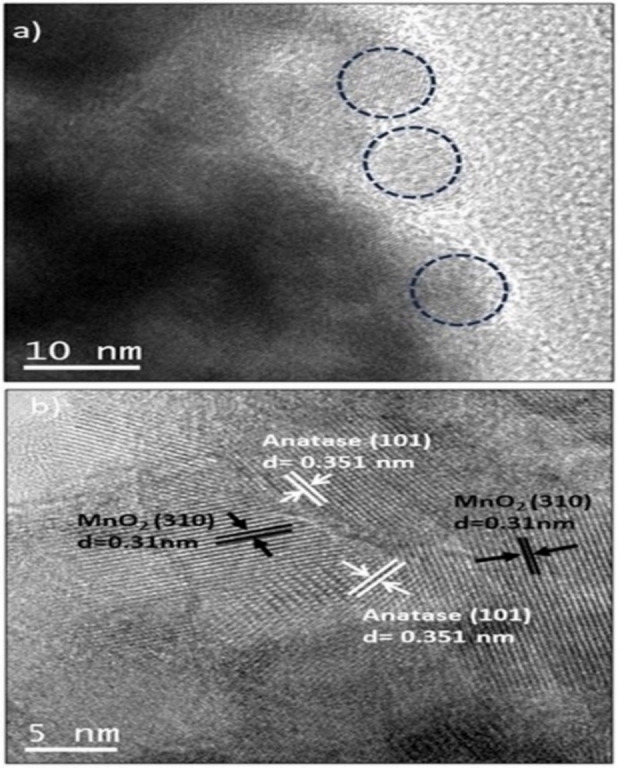
a) TEM image and b) HRTEM for 3 MnTi material.

A more detailed analysis of the photocatalyst surface was performed using the Transition Electron Microscopy (TEM) technique. Figure 3(a and b) shows the TEM images of the 3 MnTi material. In the image of Figure 3a, individual spherical‐shaped particles are observed, as well as some aggregates with a size of about 7–8 nm, which coincides with what was observed by XRD. The HR‐TEM image (Figure 3b) shows the presence of crystalline zones, which allows inferring that there is an adequate distribution of MnO_2_ throughout the material.

It is also an indication of the formation of heterojunctions between MnO_2_ and TiO_2_ particles, which was verified by calculating the interplanar distances of 0.31 and 0.35 nm, corresponding to the crystalline planes (310) of MnO_2_[^36^, ^37^] and (101) of anatase TiO_2_,^[38]^ respectively. As reported in the literature, controlled synthesis processes are responsible for promoting the formation of solid interfaces between the coupled materials, generally supported by a slow heat treatment that allows the growth of MnO_2_ particles around TiO_2_.^[34]^

The interface zones of the material also promote close contact between the semiconductors, allowing a better separation and transfer of the charge carriers, thus inhibiting recombination (*e* ^−^ *‐h* ^*+*^) and, therefore, a higher photocatalytic efficiency.

Characterization of the mesoporous nature of the samples was carried out by analyzing N_2_ adsorption‐desorption isotherms. Textural parameters, such as specific surface area and pore size distribution, were determined using the Brunauer‐Emmett‐Teller (BET) and Barrett‐Joyner‐Halenda (BJH) methods, respectively, as presented in Table 2. The results show that the TiO_2_ sol‐gel material presented a specific surface area of 76 m^2^/g. By increasing the MnO_x_ content in the materials with 0.5, 1, and 3 % MnTi, the surface areas increased to a maximum of 109 m^2^/g. This increase in the specific surface area can be attributed to an adequate dispersion of MnO_x_ on the TiO_2_ surface during the synthesis process of the nanomaterial. Increasing the surface area, also allows more organic molecules to adsorb on the semiconductor surface and react with the photogenerated electrons in the conduction band and the dissolved oxygen in the medium to produce superoxide radicals.^[39]^

**Table 2:** Morphological and optical properties of TiO_2_ and MnTi nanostructures.

Sample	BJH pore volume (cm^3^/g)	Average pore diameter (Å)	S_BET_ (m^2^/g)	Eg (eV)
TiO_2_	0.09	25	76	3.1
0.5 MnTi	0.10	24	76	2.1
1 MnTi	0.10	23	79	1.9
3 MnTi	0.11	18	109	1.8

On the other hand, it is observed that when the amount of MnO_x_ in TiO₂ is increased, the pore size decreases from 25–18 Å, similar phenomenon was also found by Min Xue et al..^[19]^ This decrease in pore size could be explained by the possible blocking of some pores in TiO₂ due to the incorporation of Mn ions during the synthesis of MnTi materials. This phenomenon results in a reduction of pore size. Moreover, this finding is consistent with the observed surface area values, which show a correlated increase with the increase in the amount of incorporated dopant.^[40]^ Although a decrease in surface area and pore volume has been documented when doping mesoporous materials,[^41^, ^42^] in this study, a slight increase in the pore volume of the synthesized materials is observed, going from 0.09–0.11 cm^3^/g. Mesoporous materials often exhibit porosities that vary in shape and size, complicating the obtaining of accurate quantitative data. In addition, in solids with extremely small pore sizes, evaporation of the condensed gas within these pores occurs with greater difficulty than condensation due to the influence of meniscus curvature.^[43]^ It has been reported that an increase in surface area and pore structure generates a synergy in electron‐hole pair formation, which is reflected in higher photocatalytic activity.^[44]^

**Figure 4:**
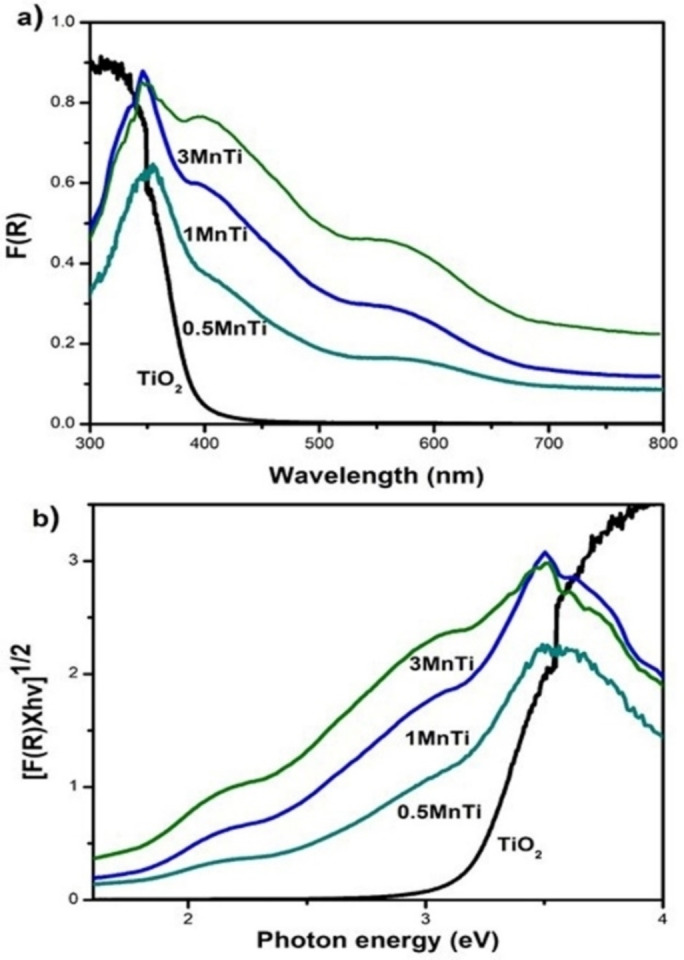
(a) Diffuse reflectance UV‐Vis spectrum and (b) Modified Kubelka‐Munk spectra of the synthesized nanomaterials.

Figure 4(a‐b) shows the UV‐visible absorption spectra for the synthesized nanomaterials. It was corroborated from the results that the doping of MnO_x_ in different percentages in the TiO_2_ matrix produces a shift towards the visible light region for all materials. In the TiO_2_ material, an absorption edge is observed with an onset near to 410 nm and an inflection point near to 370 nm; these signals are related to the Ti^+4^ matrix, indicating the presence of octahedral Ti^+4^.^[45]^ For MnTi materials, a common trend is seen in the modification of the wavelengths towards higher values and a shift of the absorption edge towards the visible region of the spectrum. These findings suggest the formation of additional electronic levels between the two semiconductors, leading to a decrease in the transition energy for the photogenerated electron‐hole pairs.

As a result, the absorption efficiency of visible light is increased.^[26]^ The bandgap energy values (Eg) of the materials were obtained from the modified Kubelka‐Munk function of the absorption spectra (Figure 4b), assuming an indirect transition for the materials, as has been reported.^[46]^ The calculated Eg values are summarized in Table 2, in which it is observed that the value of 3.1 eV (TiO_2_) was obtained much higher with respect to the values obtained for the materials 0.5 MnTi, 1 MnTi and 3 MnTi, 2.1, 1.9 and 1.8 eV, respectively.

### Photocatalytic Activity

#### Mass Loading Studies

A crucial aspect of photocatalytic processes is determining the optimal amount of photocatalytic material to avoid excess material and ensure efficient photon absorption. Excessive use of material can lead to unfavorable light scattering and reduced penetration into the solution, which is often the case when using higher concentrations of solid in photodegradation reactions. For this purpose, photocatalytic experiments were performed using different amounts of material: 50, 100, 150, and 200 mg in 200 ml of a 20 ppm solution of 2,4‐D, with an irradiation time of 360 min. Figure S1 (Supporting information) illustrates the effect of catalyst loading on 2,4‐D degradation. The results indicate that, with 50 mg, the degradation was only 10 %, while with 100 mg, this percentage increased to 21 %, more than doubling. However, with 150 and 200 mg, degradation continued to increase less significantly, reaching 25 %, a value similar to that obtained with 100 mg. Based on these observations, it was decided to opt for a minimum loading of 100 mg of catalyst in the degradation experiments to obtain acceptable degradation percentages.

All nanomaterials were evaluated in three replicates under carefully controlled conditions to ensure the accuracy and reproducibility of the photodegradation reaction of 2,4‐D herbicide under ultraviolet light irradiation. The operating conditions employed were: 200 mL solution with a concentration of 20 mg/L of 2,4‐D, a photocatalyst loading of 1 g/L, a constant air flow and UV light irradiation at 365 nm (UV Pen‐Ray lamp). The photodegradation of 2,4‐D was proceeded under UV light exposure to evaluate the photolysis reaction, i. e., in the absence of catalyst. After a period of 240 minutes, an increase in the absorbance intensity of the pollutant was observed, suggesting the formation of by‐products during this reaction. This observation confirmed that photodegradation of this pollutant did not occur in that time interval. Additionally, adsorption tests were performed on all nanomaterials in the presence of catalyst, which were carried out under dark conditions and using the experimental parameters previously described. The results of the tests, which were conducted with utmost precision, are listed in Table 3. It can be seen that as there was an increase in the MnO_2_ content, the adsorption increased in the same way from 0.0 to a maximum of 29 % for the 3 MnTi material. The photocatalytic performance of the TiO_2_ and MnTi materials was analyzed and monitored through the UV‐Vis spectroscopy technique, following the decrease in the absorption of the pollutant at λ=283 nm (S2. Supporting information). The results, which were obtained with great accuracy, are summarized in Table 3.

**Table 3:** Percentage of contaminant adsorbed in the dark and percentage of photocatalytic degradation for all nanostructures.

Sample	2,4‐D adsorbed	2,4‐D percent degraded
TiO_2_	0.0	21 %
0.5 MnTi	27 %	45 %
1 MnTi	28 %	64 %
3 MnTi	29 %	70 %

**Figure 5:**
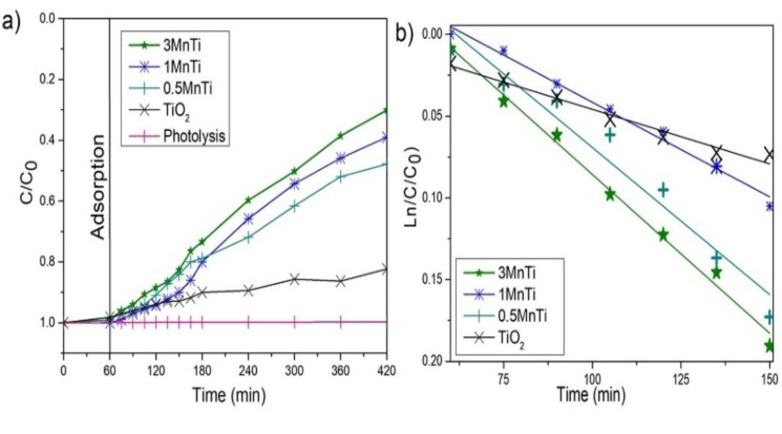
a) Photocatalytic degradation. b) Pseudo first order kinetics of 2,4‐D for TiO_2_ and MnTi nanomaterials.

According to the results obtained (Figure 5a), it was found that the TiO_2_ material decreased the concentration of the pollutant by 21 %, probably due to its rapid recombination between the generated photoelectrons and the holes. On the contrary, the photocatalytic efficiencies of all the MnTi samples were improved by the incorporation of MnO_2_, which is associated with a significant increase in the photo‐absorption of ultraviolet radiation, the possible decrease in the recombination of electron‐hole pairs, to the structural characteristics and to the synergistic interaction between the energy bands of MnO_2_ and TiO_2_. In the case of the 0.5 MnTi material, it is observed that photooxidation decays up to 45 %. However, this reduction is observed in a more pronounced way after doping with only 1 % by weight of MnO_2_, the degradation performance improved and the maximum photocatalytic efficiency achieved was 64 %. This activity was further increased by increasing the MnO_2_ content from 1 % to 3 % and obtaining a maximum degradation rate of 70 %. As could be evidenced by the analysis of surface properties, an increase in the specific surface area can increase the number of available active sites, potentially enhancing the photocatalytic activity of the compounds.^[47]^ This observation is evident in the case of the 3 MnTi material. These results show that the synthesis method used allowed easy formation of heterojunctions, which allowed increased charge dissociation in semiconductors.

From the photoactivity results, it was possible to determine the kinetic model of the pseudo first‐order reaction. Thus, from Figure 5b, it is possible to calculate the rate constant, which is also proportional to the slope of the straight line. The kinetic constant is a proportionality factor between the speed of the reaction and the concentration of the reactants; the absolute value of the constant will be higher when the catalyst presents a better photocatalytic activity. Table 4 shows that the 3 MnTi material favors a degradation 4.1 times higher than the reference material; a similar tendency presents the other two materials of the MnTi series.

**Table 4:** Kinetic parameters for photocatalytic degradation of 2,4‐D herbicide.

Sample	K(min^−1^)	t_1/2_ (min)	R^2^
TiO_2_	6.6×10^−4^	1050	0.98
0.5 MnTi	1.8×10^−3^	407	0.99
1 MnTi	1.1×10^−3^	630	0.98
3 MnTi	2.7×10^−3^	256	0.99

These results suggest that the manganese synthesis method could reduce the recombination process of the e^−^/h^+^ pair and, therefore, an improvement in the photocatalytic properties of the nanomaterials. The half‐life times (t_1/2_) presented the same trend as the kinetic constant. The calculated values were 256,630,407,1050 for 3 MnTi, 0.5 MnTi, 1 MnTi, and TiO_2_ respectively (Table 4). Finally, the partition coefficient R^2^ is also presented in Table 4.

**Figure 6:**
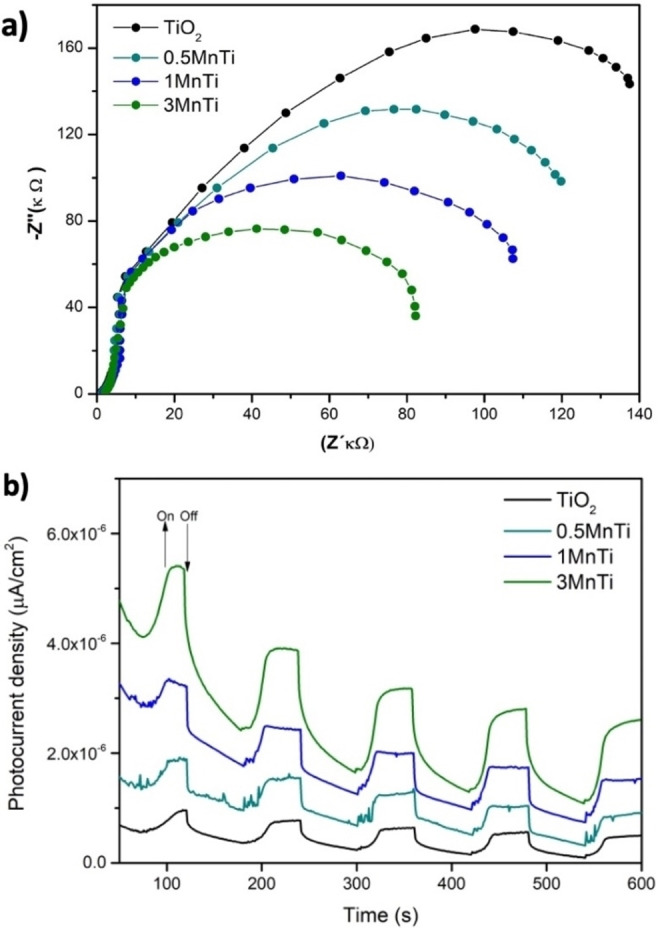
Electrochemical properties of as‐prepared samples, a) Electrochemical impedance spectroscopy (EIS) Nyquist plots and b) Photocurrent density of response (I–t) curves of TiO_2_ and MnTi photocatalyst.

To analyze the separation and migration efficiency of the charge carriers (e^−^/h^+^) in the semiconductors, photoelectrochemical measurements were performed and the results are shown in Figure 6(a‐b).

To identify the magnitude of the resistance of the synthesized samples, an EIS study was performed by applying a potential of −1 V vs Ag/AgCl, in a frequency window from 100 kHz to 1 Hz. The results were analyzed qualitatively based on the resistance obtained, considering that the magnitude of the semicircles in the EIS Nyquist plot is directly related to the rate of charge transfer measured at the electrode‐electrolyte interface. Smaller diameters indicate lower resistance to charge transfer in the semiconductors.^[33]^ It is evident that the MnTi series of semiconductors present the most minor diameters compared to the pure TiO2 sample (Figure 6a). The samples from smallest to largest diameters are 3 MnTi, 1 MnTi, 0.5 MnTi, and TiO_2_, respectively. This indicates that the MnTi series of semiconductors exhibit the lowest resistance to charge transfer and, thus, the lowest recombination rate of photogenerated charge carriers.^[44]^ A commonly employed method to determine charge separation efficiency is photocurrent. The higher the photocurrent, the higher the charge separation efficiency, which is beneficial in the photocatalytic process. To evaluate the response to transient photocurrent, on/off cycling of the lamp was performed. Figure 6b shows the photocurrent response of all the samples studied. An increase in electric current values is observed for all materials when illuminated. Subsequently, irradiation was interrupted, causing a rapid decrease in current values to zero. The transient photocurrent increased significantly in the MnTi series of materials, indicating that adding MnO2 enhanced the photocurrent response, proposing a higher photoinduced e^−^/h^+^ separation efficiency. The 3 MnTi material showed a higher current response than the rest of the materials, which affirms that there is a higher and better charge separation in 3 MnTi, which allows reacting in a more efficient way with ‐OH and, O_2_ adsorbed on the surface of the photocatalyst. These findings are consistent with the results obtained by EIS.

#### Identification of Oxidizing‐Reducing Species

**Figure 7:**
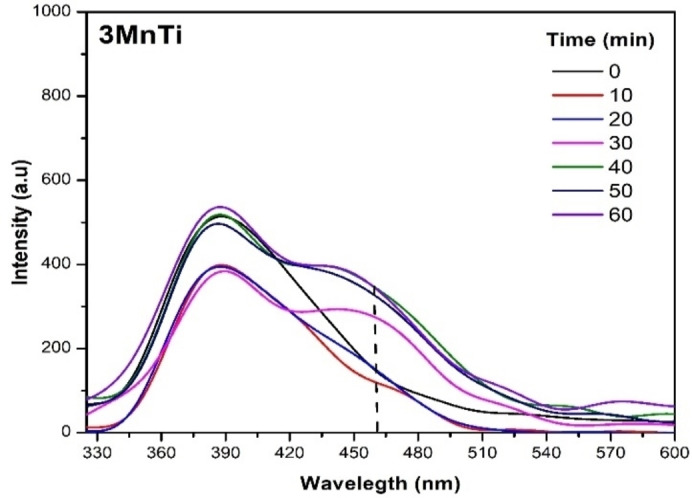
Fluorescence spectra of 7‐hydroxycoumarin for the determination of hydroxyl radicals in the 3 MnTi sample.

The study conducted to detect hydroxyl radicals (${HO}^{•}$ ), superoxide ($O_{2}^{•\;-}$ ), and hole traps ($h^{+})$ is shown below. It was carried out for the material that showed the best degradation activity, which was 3 MnTi. From the results obtained by fluorescence spectroscopy, hydroxyl radical species (${HO}^{•}$ ) were identified following the variation of 7‐hydroxycoumarin, which exhibits fluorescence in 356 nm. Tests were performed for the photolysis process, bare TiO_2_ (Supporting information) and 3 MnTi material. In the case of photolysis, a limited production of hydroxyl radicals was observed in the absence of light. In contrast, hydroxyl radical production increased up to four times when using TiO_2_, consistent with what is reported in the literature.^[48]^ However, as we can see in Figure 7, the ability to form these oxidizing species decreased significantly when using the 3 MnTi material. This result indicates that hydroxyl radicals carry out the photocatalytic degradation of 2,4‐D at a much‐reduced rate.

**Figure 8:**
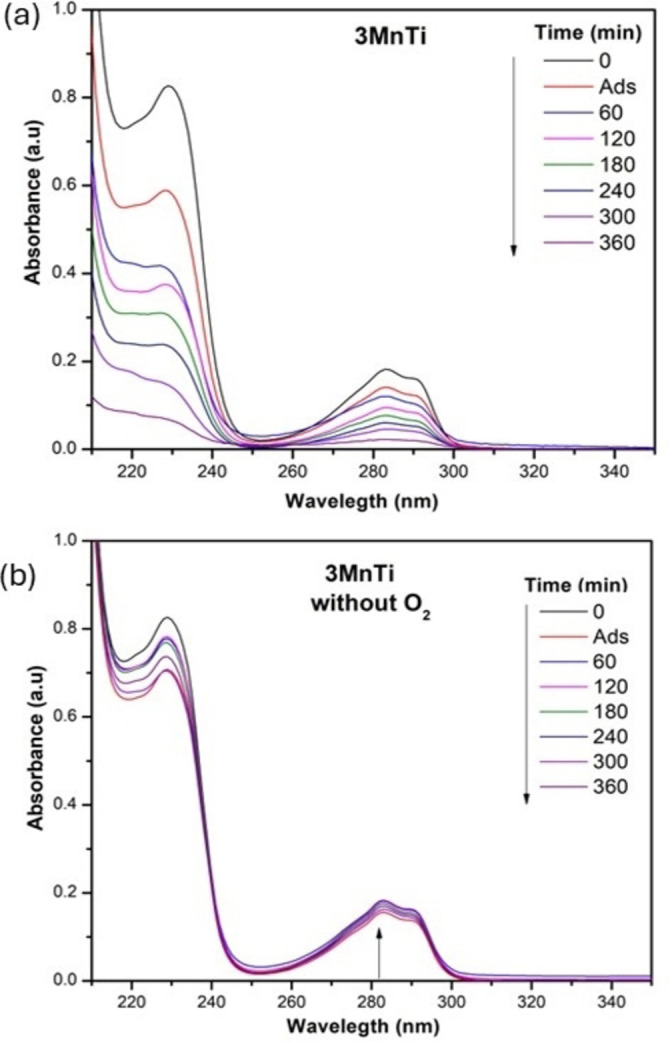
UV–Vis spectra of the photocatalytic degradation of 2,4‐D: a) suspention with O_2_; b) suspention with N_2._

On the other hand, it has been shown that $O_{2}^{•\;-}$ , generated by a reduction reaction involving electrons and oxygen from the suspention are one of the main oxidizing agents in photodegradation reactions. To understand their role in these reactions, it is necessary to remove dissolved oxygen from the water. For this purpose, before the experiments, the solution was boiled and bubbled with N_2_ and 100 mg exclusively of the 3 MnTi material was again used. The results are presented in Figure 8(a‐b). It can be observed that when the reaction was carried out suppressing the formation of $O_{2}^{•\;-}$ , the material did not present photocatalytic activity; on the contrary, a slight increase in the absorbance at 283 nm is observed, corresponding to the formation of intermediates from the 2,4‐D molecule. In contrast, when the material was under aerated conditions (Figure 8a), an evident change in the degradation of the pollutant was observed throughout the reaction time. This study supports the hypothesis that the photodegradation of 2,4‐D occurs predominantly in the presence of oxygen, which facilitates the generation of oxidative species such as the superoxide radicals.

**Figure 9:**
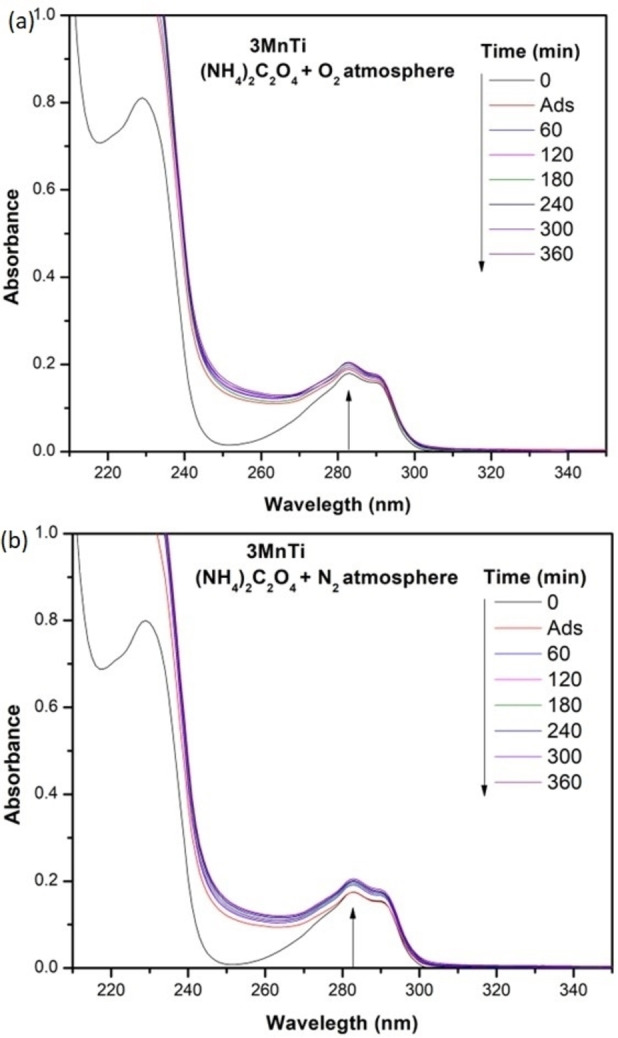
UV‐Vis spectra of the degradation of 2,4‐D in presence of ammonium oxalate: a) suspention with O_2_; b) suspention with N_2_.

During photodegradation studies, it has also been observed that using sacrificial agents helps slow down the recombination of photogenerated electron‐hole pairs, which favors the formation of hydroxyl and superoxide radicals, thus enhancing photocatalytic activity. Although most research focuses on increasing the photocatalytic activity, some studies suggest that adding hole scavengers can decrease this activity.[^49^, ^50^] Figure 9(a‐b) shows the UV‐Vis spectra of the degradation of the pollutant with ammonium oxalate. The study is presented when bubbled with air, and another is in the presence of nitrogen. As can be seen, the results of the reactions after 360 min with and without oxygen show no degradation of the pollutant, and neither is the formation of intermediates observed. Therefore, this study allowed us to determine that holes are also one of the species responsible for the photodegradation of the 2,4‐D molecule when using materials with Mn and TiO_2_ irradiated with UV light.

#### Mechanism of Photocatalytic Degradation of 2,4‐D

**Figure 10:**
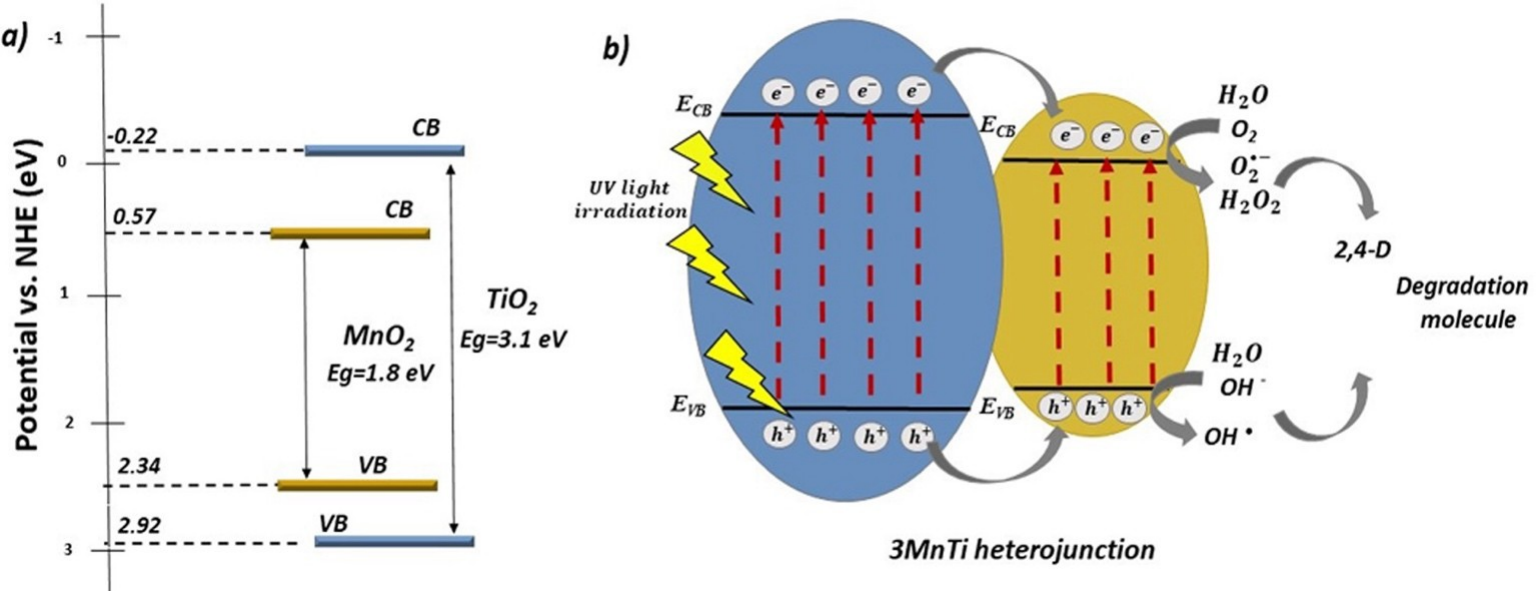
a) Relative position of the valence and conduction bands in 3 MnTi b) Possible mechanism of photocatalytic activity of the 3 MnTi heterojunction for 2,4‐D photodegradation.

In order to elucidate the charge carrier transfer mechanism, a potential reaction model for the photodegradation of the 2,4‐D herbicide under the influence of UV irradiation is proposed, as illustrated in Figure 10(a‐b). Considering the TEM results, we used Mulliken's average electronegativity theory to determine the positions of the valence band (VB) and conduction band (CB) potentials for the semiconductors TiO_2_ and MnO_2_.^[51]^ The estimated E_CB_ and E_VB_ potentials for MnO_2_ were 0.57 eV and 2.34 eV, respectively,^[52]^ while for TiO_2_, the calculated values were 2.92 eV and −0.22 eV for the conduction and valence band^[53]^ (Figure 10a). According to the estimated values, a potential mechanism has been developed for the 3 MnTi heterojunction, which demonstrated the highest photocatalytic activity. As shown in Figure 10b, the MnTi interface exhibits a type I band alignment, facilitating electron transfer from the TiO_2_ holes to the MnO_2_ conduction band and from the TiO_2_ holes to the MnO_2_ valence band. Initially, under ultraviolet irradiation, the 3 MnTi photocatalyst undergoes absorption of emitted photons, whose energy must be equal to or greater than the bandgap value of TiO_2_ (Eg=3.1 eV). Subsequently, electrons in the valence band (VB) of TiO_2_ are promoted to the vacant conduction band (CB), leading to the generation of $e^{-}/h^{+}$ pairs. Later, redox reactions are initiated between the reactive species and the 2,4‐D molecules adsorbed on the semiconductor surface. Photogenerated holes in the VB TiO_2_ can be transferred to the VB of MnO_2_, inhibiting the recombination rate and enhancing the pair $e^{-}/h^{+}$ separation processes. These photogenerated holes in the VB can react with H_2_O, producing its decomposition into 1/2O_2_ y 2H^+^ giving, rise to highly oxidizing hydroxyl radicals (${OH}^{•})$ .

In addition, the photogenerated electrons in the CB of TiO_2_ can easily migrate to the CB of MnO_2_, where they reduce the oxygen molecule, forming the superoxide radical ($O_{2}^{•-})$ . With the progress of the degradation reactions, the 2,4‐D molecules are gradually transformed into intermediates, with the eventual formation of by‐products such as CO_2_ and H_2_O. It has been observed that the inclusion of a metal oxide with a narrow forbidden bandgap, such as MnO_2_ (Eg=1.8 eV), in TiO_2_ has been shown to be beneficial in reducing the forbidden bandwidth of heterojunction and increasing the efficiency in charge separation processes. In addition, the heterojunction has increased the surface area and the number of active sites for 2,4‐D adsorption, which has facilitated the degradation process and improved the photocatalytic efficiency. These effects have been particularly prominent when using the 3 MnTi nanomaterial.^[54]^

The overall reactions of the proposed mechanism are presented below:

(1) $$hv+3MnTi\;\to \left({e_{CB}}^{-}+{h_{VB}}^{+}\right)\;3MnTi\;$$

(2) $${TiO}_{2}\;\left({h_{VB}}^{+}\right)\to {MnO}_{2}\;\left({h_{VB}}^{+}\right)$$

(3) $${TiO}_{2}\;\left({e_{CB}}^{-}\right)\to {MnO}_{2}\;\left({e_{CB}}^{-}\right)\;$$

(4) $${MnO}_{2}\;\left({e_{CB}}^{-}\right)+\;O_{2}\to \;H_{2}O_{2}$$

(5) $$H_{2}O_{2}+\;e^{-}+hv\to \;{OH}^{•}$$

(6) $$O_{2}+\;e^{-}\to \;O_{2}^{•-}$$

(7) $${MnO}_{2}\;\left({h_{VB}}^{+}\right)+\;H_{2}O\to {OH}^{•}+\;H^{+}$$

(8) $${OH}^{•}+2,4-D\to {CO}_{2}+H_{2}O$$

## Conclusions

The MnO_x_/TiO_2_ nanostructures synthesized by the sol‐gel method presented suitable optical, structural, and surface characteristics that allowed for increasing their photocatalytic activity in the degradation of the 2,4‐D herbicide under UV radiation. The 3 MnTi sample exhibited a higher yield with respect to pure TiO_2_, reaching a maximum of 70 % photooxidation after 6 hours. This increase can be attributed to different reasons: 1) the proper dispersion of MnO_x_ on the TiO_2_ surface, which allowed an increase in the contact area with the study contaminant due to the formation of smaller crystallites, 2) a greater absorption in the UV range, as well as the generation of more efficient energy states within the bandwidth, which decreased the recombination of electron‐hole pairs. Based on the electronic band structure of MnO_2_ and TiO_2_, a reasonable mechanism has been proposed for the degradation of the 2,4‐D on the 3 MnTi material, in which $O_{2}^{•\;-}$ radicals and holes stand out as the main oxidative species. Finally, based on the analyses carried out, this work indicates that this type of heterostructures could be used as a promising photocatalyst in wastewater treatment since they are low‐cost, non‐toxic materials and have the capacity to carry out adsorption and oxidation reactions at the same time.

## Experimental Section

### Reagents

The chemical reagents were used in their original form without undergoing further purification processes. Titanium (IV) isopropoxide (>98 %), isopropanol (>97 %) and nitric acid (98.9 %,

Sigma‐Aldrich) were used for the synthesis of TiO_2_. Hydrated manganese nitrate (Mn(NO_3_)_2_.H_2_O) was used as a dopant (Sigma‐Aldrich). The herbicide used in the photodegradation tests was 2,4‐dichlorophenoxyacetic acid (2,4‐D) in 99.9 % pure form, purchased from Sigma‐Aldrich. All solutions were prepared using deionized water.

### Synthesis of Heterostructures

The synthesis of the heterostructures was carried out by hydrolysis‐condensation (sol‐gel) of a titanium alkoxide using alcohol as solvent. In this procedure, titanium isopropoxide (C_12_H_28_O_4_Ti) and isopropanol (C_3_H_8_O) were used, which were mixed with 0.5 ml of nitric acid (HNO_3_) and kept at a temperature of 40 °C for a period of 2 hours (mixture 1). Measured amounts of manganese nitrite (Mn(NO_3_)_2_) were dispersed in a solution consisting of water and ethanol (mixture 2). To initiate the acid catalyzed hydrolysis process, mixture 2 was gradually added to mixture 1 over a period of 40 minutes. Once the gel was formed, it proceeded to condensation for a period of 24 hours at a temperature of 90 °C, in order to promote adequate branching of the oligomers. After this interval, the resulting wet gel was subjected to multiple washes with hot water and then dried at 90 °C for 24 hours. Finally, the solid obtained was calcined in air at a temperature of 500 °C for 4 hours, using a heating rate of 0.5 °C per minute. In this way, photocatalysts with different compositions of MnO_2_ (0.5, 1 and 3 %wt) in TiO_2_ were obtained, named 0.5 MnTi, 1 MnTi and 3 MnTi, respectively. For the preparation of bare TiO_2_, the same procedure was followed, excluding the addition of the MnO_2_ precursors.

### Characterization

The study of morphology and particle size of the samples were carried out by scanning electron microscopy (SEM) using a JEOL model JSM‐6390LV, operating at an accelerating voltage of 15 kV. Quantitative determination of the element composition present in the samples was carried out using X‐ray energy dispersive spectroscopy (EDS). Transmission electron microscopy (TEM) analysis was performed using a JEOL 2100F HRTEM machine, with an accelerating voltage of 200 Kv. X‐ray diffraction (XRD) patterns were characterized using a Bruker D2 PHASER X‐ray diffraction spectrophotometer with a Cu−Kα radiation source (λ=1.54 Å) operating at 40 kV, with a scan rate of 0.3 s per step, over a range of 10° to 70° (2θ angle) Crystallite size estimation was calculated using the Scherrer equation from the data obtained. The analysis of the textural properties of the nanomaterials was carried out by means of adsorption‐desorption isotherms using a Quantachrome equipment, model NOVA. Prior to analysis, all samples were degassed under vacuum at a temperature of 250 °C for 15 hours. Diffuse reflectance spectroscopy (DRS) was performed using a Varian Cary 100 UV‐visible DRS spectrometer. Determination of the energy values of the forbidden band (Eg) was performed using the Kubelka‐Munk equation. The electrochemical analyses were performed employing a potentiostat/galvanostat AUTOLAB model PGSTAT302 N with FRA module. The identification of hydroxyl (${HO}^{•}$ ) radicals was measured by fluorescence probe, using a SCINCO FS‐2 fluorescence spectrometer, with at wavelength of λ=332 nm.

### Photodegradation Process of 2,4‐D

The efficiency of the nanomaterials synthesized was measured by the photocatalytic degradation of the 2,4‐D molecule (20 ppm) in aqueous solution under UV irradiation. The tests were carried out in a recirculation system, which consists of a glass reactor (200 mL) surrounded by a thermostatic glass jacket (temperature 25 °C). The suspension was placed between the glass reactor and the immersion cell containing the lamp (365 nm at 4400 μW/cm^2^). Then 100 mg of MnTi nanomaterials were added for each test. The resulting suspension was bubbled for 60 minutes in order to achieve adequate dispersion and establish the adsorption‐desorption equilibrium of the 2,4‐D herbicide at the active sites on the surface of the heterostructure. After this period, the UV lamp was turned on and aliquots were taken at 30 min intervals for 4 hours. The herbicide concentration was determined using a UV‐Vis spectrophotometer, observing the characteristic signal of 2,4‐D at a wavelength of 283 nm. All experiments were repeated several times and the results were averaged.

The determination of hydroxyl radicals involves the addition of 1 g/L of to a 2×10^−3^ M coumarin solution. The suspension was maintained under precise conditions: agitation (600 rpm), air conditions (1 mL s^−1^), and a UV lamp (Pen UV‐Ray, λ=365 nm, 4 mW/cm^2^). Aliquots were taken every 10 min and analyzed by photoluminescence using an excitation wave of 332 nm. For the superoxide radicals experiments, 1 g/L of the most active photocatalyst was added to an aqueous solution containing 20 ppm of 2,4‐D. The suspension was kept for one hour under bubbling conditions with N_2_ (at 1 mL s^−1^) and stirring (600 rpm). After that time, the reaction was initiated by irradiating with the same UV lamp used for hydroxyl radicals. Photodegradation was monitored for 360 min by UV‐Vis spectroscopy. To determine the impact of holes on the degradation of organic compounds, the reduction in the percentage of photodegradation in the presence of ammonium oxalate as a sacrificial agent was measured. In this study, the degradation of 2,4‐D was examined under the same conditions described in the photodegradation process of 2,4‐D section, using a 4×10^−3^ M ammonium oxalate concentration. The presence of holes was determined in experiments with O_2_ bubbling and the presence of N_2_ only for the 3 MnTi material. The reaction was carried out for 360 min, taking aliquots at 1 h intervals and measuring their activity by UV‐Vis spectroscopy.

## Conflict of Interests

The authors declare that they have no known competing financial interests or personal relationships that could have appeared to influence the work reported in this paper.

1

## Supporting information

As a service to our authors and readers, this journal provides supporting information supplied by the authors. Such materials are peer reviewed and may be re‐organized for online delivery, but are not copy‐edited or typeset. Technical support issues arising from supporting information (other than missing files) should be addressed to the authors.

Supporting Information

## Data Availability

No data was used for the research described in the article.
