# Supplementary material for: Design of MnOx/TiO2 Nanostructures for Photocatalytic Removal of 2,4‐D Herbicide
Source: ChemistryOpen. 2024 Dec 13;14(1):e202400154. doi: 10.1002/open.202400154 (PMC11726695; doi:10.1002/open.202400154)
Supplement: Supplementary file 1 — Supporting Information [file OPEN-14-e202400154-s001.pdf]

ChemistryOpen

Supporting Information

Design of MnO_x/TiO_2 Nanostructures for Photocatalytic Removal of 2,4-D Herbicide

Angeles Mantilla, Sandra Cipagauta Díaz, Enrique Samaniego Benitez, Francisco Javier Tzompantzi Morales, and Michelle Navarrete Magaña*

Supporting information

Design of MnO_x/TiO_2 Nanostructures for Photocatalytic Removal of 2,4-D Herbicide

Angeles Mantilla[a], Sandra Cipagauta Díaz[b], Enrique Samaniego Benitez[c], Francisco Javier Tzompantzi Morales[b], Michelle Navarrete Magaña*[a,d]

[a] Dr. A. Mantilla, Dr. M. Navarrete Magaña
Advanced Technology
Instituto Politécnico Nacional, CICATA-Legaria, Legaria 694, Col. Irrigación, 11500, Ciudad de México
E-mail: anavarretem@ipn.mx

[b] Dr. S. Cipagauta Díaz, Dr. F.J. Tzompantzi Morales
Chemistry deparment
Universidad Autónoma Metropolitana-Iztapalapa, Av. San Rafael Atlixco 186, Leyes de Reforma, 09340, Ciudad de México

[c] Dr. E. Samaniego Benitez
Avanced Technology
CONAHCyT-Instituto Politécnico Nacional, CICATA-Legaria, Legaria 694, Col. Irrigación, 11500, Ciudad de México

[d] Dr. M. Navarrete Magaña
Chemistry academies
Instituto Politécnico Nacional, UPIICSA, Av. Té 950, Granjas México, 08400 Ciudad de México

Mass loading studies

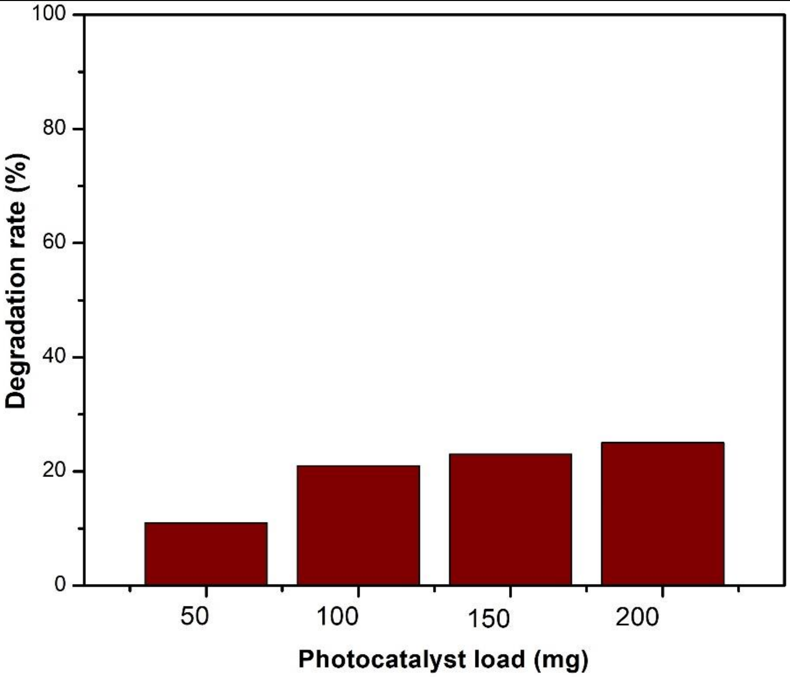

Figure S1.- Effect of the TiO_2 loading on the photodegradation of 2,4-D.

Photocatalytic degradation of 2,4-D

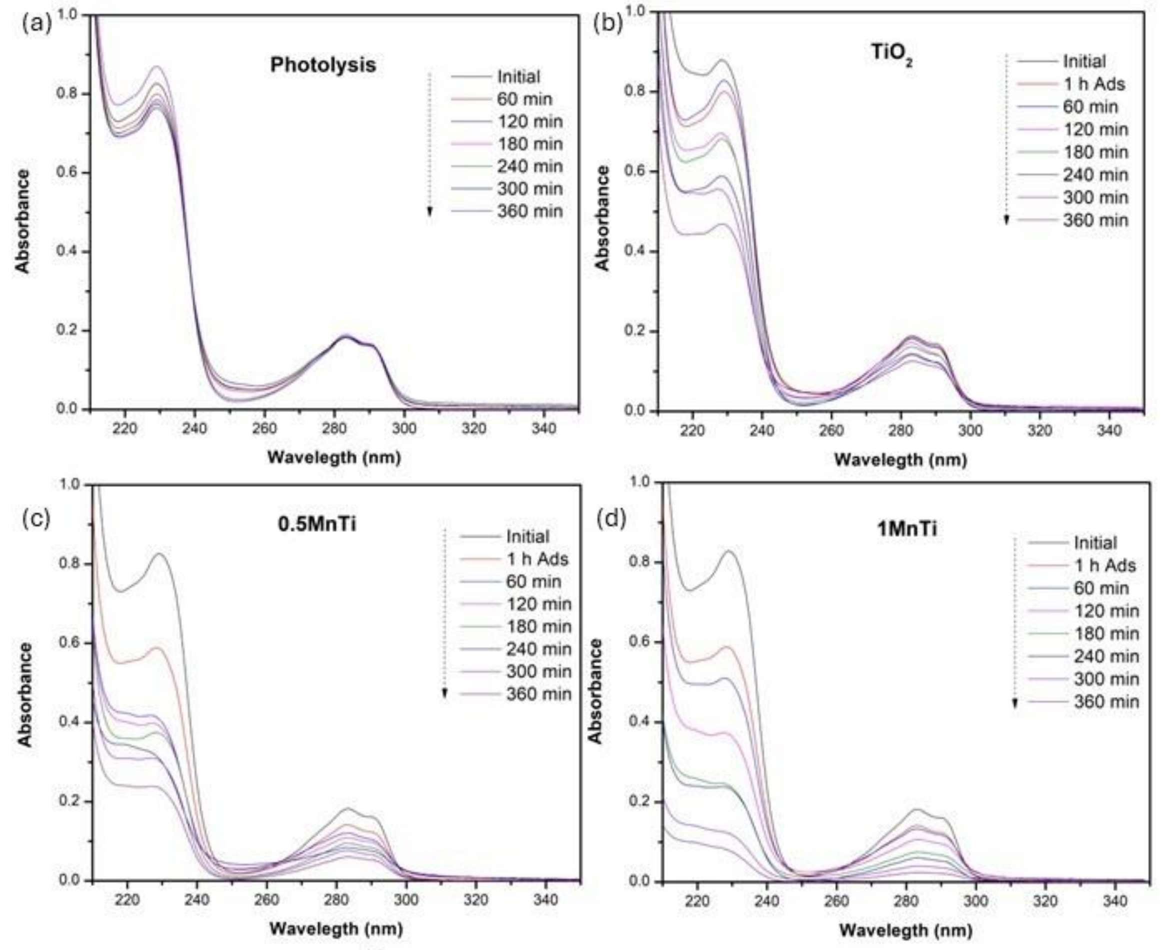

Figure S2.- Photodegradation absorption spectra of the 2,4-D solution under irradiation with ultraviolet light in the photolysis experiment (a), and with the TiO_2 (b), 0.5MnTi (c), and 1MnTi photocatalysts.

Hydroxyl radicals' determination

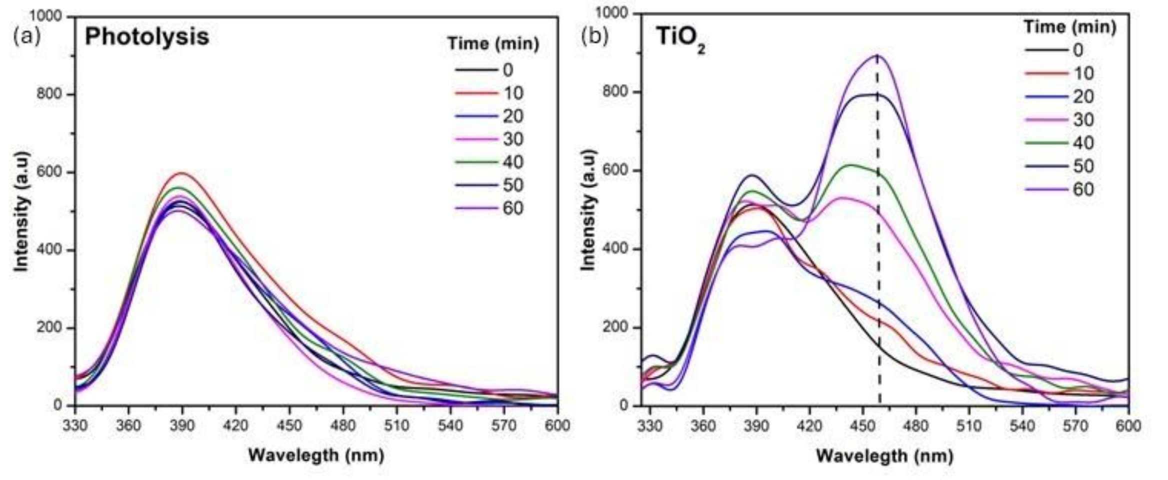

Figure S3.- Fluorescence spectra of 7-hydroxycoumarin for the determination of hydroxyl radicals in the photolysis and TiO_2 experiments.
